# USE OF A BRAIN CARE SCORE TO IDENTIFY RISK AND PROTECTIVE FACTORS FOR MAINTAINING COGNITION AND HEALTH IN LATER LIFE

**DOI:** 10.1093/geroni/igae098.3054

**Published:** 2024-12-31

**Authors:** Kylie Schiloski, Morgan Taylor, Margie Lachman

**Affiliations:** Brandeis University, Waltham, Massachusetts, United States; Brandeis University, Waltham, Massachusetts, United States; Brandeis University, Waltham, Massachusetts, United States

## Abstract

The Massachusetts General Hospital McCance Center for Brain Health recently developed a Brain Care Score (BCS) to assess the risk of dementia and stroke and to promote healthier lifestyles. The BCS includes a combination of modifiable physical (e.g., blood pressure, blood glucose, cholesterol, Body Mass Index), environmental (e.g., nutrition, exercise, smoking status, sleep, alcohol consumption), and psychosocial factors (e.g., perceived stress, social support, purpose in life) that have been shown to predict dementia and stroke. The goal of the present study was to develop and test a comparable measure using data from the Midlife in the United States study (MIDUS). We examined whether the MIDUS version of the BCS predicted changes in cognition and health from the second (M2; 2004-2005) to the third wave (M3; 2013-2014) of the Biomarker sub-study sample (M2: N=1,167; Mage=54.46; SD=11.60; Age Range=34-83). Multiple regressions showed that higher BCSs predicted less decline in episodic memory (immediate and delayed recall), fluid intelligence (number series), processing speed, (backwards counting), and verbal fluency (category fluency) over time as well as less decline in functional health and fewer chronic conditions after 8 to 10 years. These findings indicate that the MIDUS version of the BCS is a promising tool to help identify those at risk of declines in cognition and health. Moreover, these findings highlight the importance of adopting a healthy lifestyle in order to reduce one’s risk of experiencing declines in cognitive and physical health in middle and later adulthood.

